# An Account of a Rupture of the Pylorus, with the Appearances after Death

**Published:** 1828-06

**Authors:** 


ART. II.—*An account of a rupture of the Pylorus, with the appearances after death.* BY THE EDITOR.

In the 10th number of the Western Medical and Physical Journal, I published an account of the death of John McDonald, from a rupture in the Jejunum from external violence, the abdominal parieties remaining entire. I am now about to report a case of ruptured Pylorus, attended with analagous symptoms, and terminating in a similar manner.

In the afternoon of the 14th of April, 1828, I was called to see E. P. aged 38 years, by occupation a cordwainer. He was said to labour under colic; and to have been attacked suddenly and violently. I found him sitting up, with a pale and cool skin, a feeble pulse, beating not more than 48 strokes in a minute, and complaining of excruciating spasmodic pain in the abdomen, the paroxysms of which were followed by incomplete fainting fits. Regarding it as an attack of flatulent colic, I, immediately, directed a hot, foot-bath; and began the preparation of a large dose of opium and calomel. Before it was ready for administration, he was seized with retchings, and drank a glass of tepid water. Slight vomiting occurred, with agonizing pain, which was followed by a degree of syncope that bordered on dissolution. As soon as he revived the portion of opium and calomel was administered, together with a quantity of paregoric. Stimulating, cathartic enemas were then employed; but they produced no fæcal evacuation, and, indeed, were themselves but partially discharged. About 10 o'clock I left him for the night, in the care of my young friend Dr. McDowell. The calomel and opium were repeated; laudanum was liberally administered; a dose of castor oil was given, and the injections were repeated, in the course of the night; but nothing more than palliation followed. The next morning, I found him thirsty, in pain and drowsy, with his abdomen distended, especially *in epigastrio*, and tender on pressure. His pulse was feeble, exceedingly variable in its rythm, at no time more than 60 in a minute, and frequently down to 46. It was obvious that peritonitis had supervened, and I bled him. The blood was sizy; but his pulse fell instead of rising in its energies; no alvine evacuation ensued; nor were the pain and swelling diminished. Calomel was now administered in large and repeated portions, with occasional doses of sulphate of magnesia and castor oil; the injections were repeated, and the abdomen was blistered. In the evening the symptoms were mitigated, and a small quantity of feculent matter was discharged. During the night a gentle salivation came on; he rested badly; had occasional retchings, in which he threw up but little; and the next morning was worse, than I had left him in the evening. The epigastric tension, and soreness on pressure, especially, had increased. The pulse remained nearly the same in frequency, but was still weaker and smaller. The case now gave me equal embarrassment and apprehension; for it exhibited unequivocal evidences of abdominal inflammation, combined with an exhaustion of the vital powers, which was as manifestly occasioned by something else, than the peritonitis, which had been developed. I could not doubt that a structural læsion existed; but of what kind it seemed impossible to determine. Examination was made for hernia—ventral, inguinal and femoral—but none existed; and I was led to conjecture that intussusceptio had taken place. I desired a consultation, and, in the afternoon, Dr. Pierson was called in. He was struck with the obvious signs of peritoneal inflammation; and the patient was again bled, more copiously than before. The pulse sunk during the operation, and the blood was not sizy. The striking resemblance of the case to that of McDonald, confirmed me in the opinion of its necessary mortality; but, as the patient retained his senses perfectly, and with his friends was desirous of being engaged in curative measures of some kind, they were continued. Among other means the croton oil and ol: teribinth: were given liberally,—the latter was applied to the abdomen, and tobacco injections were administered: but every thing proved unavailing. The abdominal tumefaction and agony increased; his pulse became imperceptible, and towards morning he expired; about 60 hours from the commencement of the attack.

### EXAMINATION OF THE ABDOMEN.

Leave being granted to inspect the abdomen, it was done the evening after his death. The distension was very considerable, and upon penetrating the cavity of the peritoneum, a great quantity of offensive gas escaped. On making an extended crucial incision, that membrane was found to be inflamed, and to have thrown out a quantity of coagulable lymph, with some admixture of pus. It was slightly agglutinated to the bowels; and these were extensively adherent to each other. The whole were immersed in a turbid liquor, of a yellowish colour, which emitted the smell of turpentine, and exhibited no inconsiderable quantity of oil floating on its surface. A large worm (lumbricoides) was crawling over the bowels. After sponging out between two and three quarts of fluid, (the quantity extravasated) we found the outer tunic of all the anterior and upper convolutions of the intestines, great and small, in a state of inflammation. The omentum was in the same condition. The spleen was large, tender, and so much inflamed, that its peritoneal covering peeled off. The liver was but little affected. The gall bladder was distended with healthy bile. The urinary bladder was empty and uninflamed. On examining the intestines, from the rectum to the stomach, no læsion was found, till we reached the pylorus; where the cause of all the mischief was discovered. *It was ruptured more than half way round, and the fissure exhibited the appearance of a clean cut.* Through this, the contents of the stomach and duodenum had escaped into the cavity of the peritoneum. On slitting open the bowels and stomach, the mucous membrane of the former was healthy, and the pyloric extremity of the stomach was equally healthy, on both sides. The mucous coat of its great extremity was considerably, but not deeply inflamed. No ulceration existed any where; nor had any part suffered gangrene. The closest inspection of the pylorus, did not enable us to detect any organic change, that could account for its laceration.

REMARKS.

I shall not indulge in many speculations on this case. Two or three sentences may contain all that I have to say. The deceased had labored at his vocation, with unabated diligence throughout the day, and was occupied, in a way that required him to press the end of a board against the pit of his stomach, or near that part. This was the only external violence that he suffered. At one o'clock he ate a hearty dinner; and between four and five o'clock was suddenly seized with the pain that has been described. Two hours afterwards, in attempting to vomit, he experienced the insupportable agony with syncope, which has been mentioned in the history of his case. Now the question arises, whether the rupture of the Pylorus preceded and occasioned the other symptoms, or occurred in the efforts to vomit?

Under either supposition, we may conjecture that his daily labours may have, to a certain extent, impaired the health and firmness of his epigastric viscera; and, consequently, predisposed them to suffer a læsion of structure. His stomach seems to have been torpid; for, when he vomited, he threw up undigested food which had been eaten two days before. Perhaps, therefore, it was greatly distended with flatus, while the Pylorus was contracted, as it is for the few first hours after dinner; in which condition the external pressure on the epigastrium might have produced the laceration. If it did not happen in this way, it would seem to have been caused by the vomiting.

